# μ-Oxalato-bis­[bis­(triphenyl­phosphine)copper(I)] dichloro­methane disolvate^1^

**DOI:** 10.1107/S1600536813002080

**Published:** 2013-01-31

**Authors:** Andrew D. Royappa, James A. Golen, Arnold L. Rheingold, A. Timothy Royappa

**Affiliations:** a Department of Chemistry, University of West Florida, 11000 University Parkway, Pensacola, FL 32514, USA; b Department of Chemistry, University of Massachusetts Dartmouth, 285 Old Westport Road, North Dartmouth, MA 02747, USA; c Department of Chemistry, University of California, San Diego, Urey Hall 5128, mail code 0358, 9500 Gilman Drive, La Jolla, CA 92093, USA

## Abstract

The dinuclear molecule of the title compound, [Cu_2_(C_2_O_4_)(C_18_H_15_P)_4_]·2CH_2_Cl_2_, lies across an inversion center with a strictly planar bridging oxalate ligand coordinating two Cu^I^ ions *via* two pairs of O atoms. Two triphenyl­phosphine ligands also coordinate each symmetry-related Cu^I^ ion, resulting in a distorted tetra­hedral geometry [O—Cu—O = 80.57 (5)° and P—Cu—P = 125.72 (2)°]. In the crystal, there are two dichloro­methane solvent mol­ecules for each dinuclear complex.

## Related literature

For the applications of copper(I) oxalates, see: Doyle (1982 ▶); Köhler *et al.* (2003 ▶); Angamuthu *et al.* (2010 ▶). For a comprehensive patent covering CVD applications of copper(I) oxalates, see: Köhler & Meyer (2004 ▶). For related copper(I) oxalate complexes, see: Frosch *et al.* (2000 ▶); He *et al.* (2008 ▶); Teichgräber *et al.* (2005 ▶). For the chemical fixation of CO_2_ to form oxalates, see: Savéant (2008 ▶). For an alternate synthesis of the title compound, see: Díez *et al.* (1988 ▶).
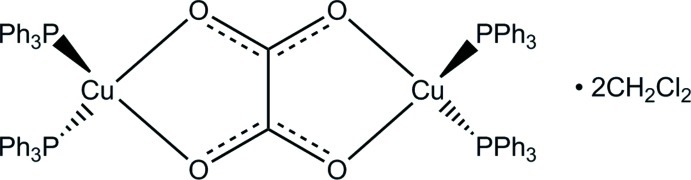


## Experimental

### 

#### Crystal data

[Cu_2_(C_2_O_4_)(C_18_H_15_P)_4_]·2CH_2_Cl_2_
*M* *_r_* = 1434.03
Monoclinic, 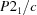

*a* = 13.4735 (4) Å
*b* = 14.7294 (4) Å
*c* = 18.2282 (6) Å
β = 109.255 (1)°
*V* = 3415.14 (18) Å^3^
*Z* = 2
Mo *K*α radiation
μ = 0.92 mm^−1^
*T* = 100 K
0.30 × 0.25 × 0.20 mm

#### Data collection

Bruker Kappa diffractometer equipped with a Photon100 CMOS detector
Absorption correction: multi-scan (*SADABS*; Bruker, 2007 ▶) *T*_min_ = 0.769, *T*_max_ = 0.837
27318 measured reflections
6960 independent reflections
5738 reflections with *I* > 2σ(*I*)
*R*_int_ = 0.053

#### Refinement

*R*[*F* ^2^ > 2σ(*F* ^2^)] = 0.035
*wR*(*F* ^2^) = 0.088
*S* = 1.01
6960 reflections
406 parameters
H-atom parameters constrained
Δρ_max_ = 0.83 e Å^−3^
Δρ_min_ = −0.55 e Å^−3^

### 

Data collection: *APEX2* (Bruker, 2007 ▶); cell refinement: *SAINT* (Bruker, 2007 ▶); data reduction: *SAINT*; program(s) used to solve structure: *SHELXS97* (Sheldrick, 2008 ▶); program(s) used to refine structure: *SHELXL97* (Sheldrick, 2008 ▶); molecular graphics: *PLATON* (Spek, 2009 ▶); software used to prepare material for publication: *SHELXTL* (Sheldrick, 2008 ▶) and *CHEMDRAW* (Cambridgesoft, 2003 ▶).

## Supplementary Material

Crystal structure: contains datablock(s) I, global. DOI: 10.1107/S1600536813002080/lh5575sup1.cif

Structure factors: contains datablock(s) I. DOI: 10.1107/S1600536813002080/lh5575Isup2.hkl

Supplementary material file. DOI: 10.1107/S1600536813002080/lh5575Isup3.cdx

Additional supplementary materials:  crystallographic information; 3D view; checkCIF report

## Figures and Tables

**Fig. 1.**
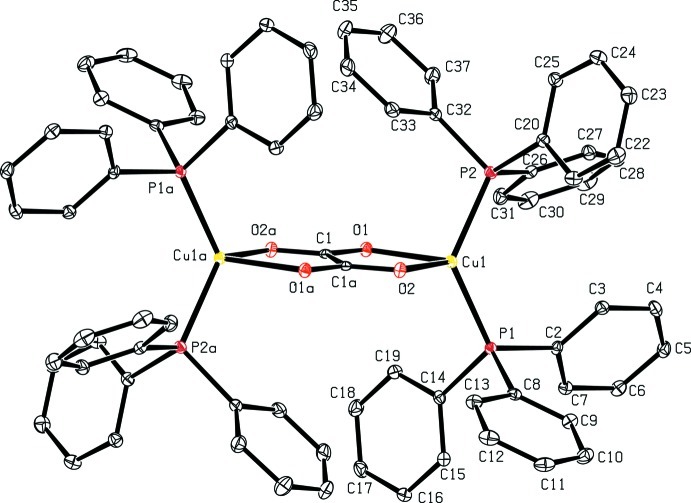
The molecular structure of the title compound showing displacement ellipsoids at the 30% probability level. Neither the H atoms nor the solvent molecules are shown (Symmetry code: (a) -x, -y+1, -z+1).

## supplementary crystallographic information

### Comment

Though numerous copper(II) oxalate complexes are known, copper(I) oxalates as a family are poorly understood. Nevertheless, the latter compounds have important applications in, *e.g.*, CO capture (Doyle, 1982), CVD of metallic copper (Köhler *et al.*, 2003; Köhler & Meyer, 2004) and CO_2_ fixation (Angamuthu *et al.*, 2010). Very few oxalato complexes of copper(I) have been structurally characterized, and those that have been studied crystallographically are organometallic species containing ligands bound to the copper(I) centers *via* carbon atoms (Köhler *et al.*, 2003; Teichgräber *et al.*, 2005). To date, no examples of copper(I) oxalate compounds containing triphenylphosphine ligands coordinated through the phosphorus atoms to the metal centers have been structurally characterized.

The molecular structure of the title compound is shown in Fig. 1. The dinuclear complex lies across an inversion center. In addition, the asymmetric unit contains a dichloromethane solvent. The Cu^I^ ions are bridged by a strictly planar oxalate ligand, with two oxygen atoms coordinated to each Cu^I^ ion. The coordination geometry at each Cu^I^ ion is distorted tetrahedral. The bite angle involving the oxalate ligand is fairly small (80.57 (5)°), while the two phosphorus atoms from the coordinated triphenylphosphine ligands form an angle of 125.72 (2)° with each symmetry-related Cu^I^ ion. A similar geometry is observed in the copper(I) oxalate isonitrile complexes studied previously (Teichgräber *et al.*, 2005).

### Experimental

All manipulations were carried out on a Schlenk line under nitrogen unless otherwise mentioned. Initially, bis(tetrabutylammonium) oxalate was synthesized *in situ* by dissolving 1 ml of 1 *M* tetrabutylammonium hydroxide solution (in methanol; 1 mmol) and 0.045 g anhydrous oxalic acid (0.5 mmol) in 20 ml degassed absolute ethanol. Next, 0.526 g triphenylphosphine (2 mmol) were dissolved in this solution. Separately, 0.373 g tetrakis(acetonitrile)copper(I) hexafluorophosphate (1 mmol) were dissolved in 20 ml degassed absolute ethanol to form a cloudy solution, which was added to the oxalate solution. The product was formed by the metathesis reaction as a white precipitate, washed with 3 *x* 5 ml ice-cold degassed absolute ethanol, dried under a nitrogen stream and finally air-dried. Colorless block crystals were grown at room temperature from dichloromethane by layering with hexane under nitrogen. The title compound has also been prepared by Díez *et al.* (1988) by an alternate method.

### Refinement

H atoms were placed in calculated positions with C—H = 0.95Å (phenyl) or C—H = 0.96Å (solvent CH_2_) and included in the refinement in a riding-motion approximation with U_iso_(H) = 1.2U_eq_(C).

### Crystal data

**Table:**

[Cu_2_(C_2_O_4_)(C_18_H_15_P)_4_]·2CH_2_Cl_2_	*F*(000) = 1476
*M**_r_* = 1434.03	*D*_x_ = 1.395 Mg m^−^^3^
Monoclinic, *P*2_1_/*c*	Mo *K*α radiation, λ = 0.71073 Å
Hall symbol: -P 2ybc	Cell parameters from 8477 reflections
*a* = 13.4735 (4) Å	θ = 2.7–26.4°
*b* = 14.7294 (4) Å	µ = 0.92 mm^−^^1^
*c* = 18.2282 (6) Å	*T* = 100 K
β = 109.255 (1)°	Block, colourless
*V* = 3415.14 (18) Å^3^	0.30 × 0.25 × 0.20 mm
*Z* = 2	

### Data collection

**Table:**

Bruker Kappa diffractometer equipped with a Photon100 CMOS detector	6960 independent reflections
Radiation source: high-brilliance IµS microsource	5738 reflections with *I* > 2σ(*I*)
Doubly curved mirrors monochromator	*R*_int_ = 0.053
φ and ω scans	θ_max_ = 26.4°, θ_min_ = 2.7°
Absorption correction: multi-scan (*SADABS*; Bruker, 2007)	*h* = −16→16
*T*_min_ = 0.769, *T*_max_ = 0.837	*k* = −18→18
27318 measured reflections	*l* = −22→21

### Refinement

**Table:**

Refinement on *F*^2^	Primary atom site location: structure-invariant direct methods
Least-squares matrix: full	Secondary atom site location: difference Fourier map
*R*[*F*^2^ > 2σ(*F*^2^)] = 0.035	Hydrogen site location: inferred from neighbouring sites
*wR*(*F*^2^) = 0.088	H-atom parameters constrained
*S* = 1.01	*w* = 1/[σ^2^(*F*_o_^2^) + (0.0382*P*)^2^ + 2.6822*P*] where *P* = (*F*_o_^2^ + 2*F*_c_^2^)/3
6960 reflections	(Δ/σ)_max_ = 0.002
406 parameters	Δρ_max_ = 0.83 e Å^−^^3^
0 restraints	Δρ_min_ = −0.55 e Å^−^^3^

### Special details

**Table:**

Geometry. All e.s.d.'s (except the e.s.d. in the dihedral angle between two l.s. planes) are estimated using the full covariance matrix. The cell e.s.d.'s are taken into account individually in the estimation of e.s.d.'s in distances, angles and torsion angles; correlations between e.s.d.'s in cell parameters are only used when they are defined by crystal symmetry. An approximate (isotropic) treatment of cell e.s.d.'s is used for estimating e.s.d.'s involving l.s. planes.
Refinement. Refinement of *F*^2^ against ALL reflections. The weighted *R*-factor *wR* and goodness of fit *S* are based on *F*^2^, conventional *R*-factors *R* are based on *F*, with *F* set to zero for negative *F*^2^. The threshold expression of *F*^2^ > σ(*F*^2^) is used only for calculating *R*-factors(gt) *etc*. and is not relevant to the choice of reflections for refinement. *R*-factors based on *F*^2^ are statistically about twice as large as those based on *F*, and *R*- factors based on ALL data will be even larger.

### Fractional atomic coordinates and isotropic or equivalent isotropic displacement parameters (Å^2^)

**Table:**

	*x*	*y*	*z*	*U*_iso_*/*U*_eq_	
Cu1	0.125878 (19)	0.366123 (17)	0.583149 (14)	0.01365 (8)	
P1	0.07910 (4)	0.22305 (4)	0.55130 (3)	0.01249 (12)	
P2	0.28337 (4)	0.41243 (4)	0.65979 (3)	0.01340 (12)	
O1	0.10808 (11)	0.44751 (10)	0.48437 (8)	0.0150 (3)	
O2	−0.00215 (11)	0.44403 (10)	0.58463 (8)	0.0159 (3)	
C1	0.03183 (15)	0.50110 (14)	0.47093 (11)	0.0122 (4)	
C2	0.18062 (16)	0.13501 (14)	0.57999 (11)	0.0137 (4)	
C3	0.25280 (17)	0.13802 (15)	0.65523 (12)	0.0171 (4)	
H3A	0.2519	0.1878	0.6881	0.021*	
C4	0.32562 (17)	0.06921 (16)	0.68236 (12)	0.0210 (5)	
H4A	0.3737	0.0716	0.7339	0.025*	
C5	0.32864 (17)	−0.00325 (15)	0.63458 (13)	0.0207 (5)	
H5A	0.3779	−0.0510	0.6535	0.025*	
C6	0.25906 (17)	−0.00560 (15)	0.55883 (13)	0.0193 (5)	
H6A	0.2621	−0.0543	0.5255	0.023*	
C7	0.18551 (17)	0.06262 (14)	0.53183 (12)	0.0158 (4)	
H7A	0.1379	0.0602	0.4801	0.019*	
C8	−0.01994 (16)	0.17979 (14)	0.59146 (11)	0.0149 (4)	
C9	−0.01072 (18)	0.09664 (15)	0.62985 (12)	0.0189 (5)	
H9A	0.0494	0.0597	0.6366	0.023*	
C10	−0.08918 (19)	0.06765 (16)	0.65827 (13)	0.0247 (5)	
H10A	−0.0819	0.0114	0.6850	0.030*	
C11	−0.17783 (19)	0.12024 (16)	0.64786 (13)	0.0244 (5)	
H11A	−0.2318	0.0999	0.6668	0.029*	
C12	−0.18743 (18)	0.20289 (17)	0.60956 (13)	0.0246 (5)	
H12A	−0.2485	0.2389	0.6019	0.029*	
C13	−0.10859 (17)	0.23327 (15)	0.58236 (12)	0.0197 (5)	
H13A	−0.1150	0.2907	0.5575	0.024*	
C14	0.01648 (16)	0.20439 (14)	0.44683 (11)	0.0141 (4)	
C15	−0.05785 (17)	0.13649 (15)	0.41739 (12)	0.0179 (4)	
H15A	−0.0780	0.0982	0.4520	0.021*	
C16	−0.10276 (19)	0.12437 (15)	0.33775 (12)	0.0219 (5)	
H16A	−0.1545	0.0787	0.3180	0.026*	
C17	−0.07221 (19)	0.17883 (16)	0.28711 (12)	0.0240 (5)	
H17A	−0.1021	0.1698	0.2326	0.029*	
C18	0.00167 (19)	0.24642 (16)	0.31567 (13)	0.0243 (5)	
H18A	0.0226	0.2836	0.2807	0.029*	
C19	0.04551 (17)	0.26022 (15)	0.39536 (12)	0.0179 (4)	
H19A	0.0951	0.3076	0.4148	0.021*	
C20	0.31334 (16)	0.41751 (14)	0.76456 (11)	0.0148 (4)	
C21	0.25409 (18)	0.36501 (15)	0.79846 (13)	0.0203 (5)	
H21A	0.1967	0.3301	0.7666	0.024*	
C22	0.27853 (19)	0.36349 (16)	0.87860 (13)	0.0241 (5)	
H22A	0.2380	0.3274	0.9014	0.029*	
C23	0.36174 (19)	0.41440 (16)	0.92545 (12)	0.0226 (5)	
H23A	0.3788	0.4127	0.9803	0.027*	
C24	0.41993 (18)	0.46772 (16)	0.89227 (12)	0.0221 (5)	
H24A	0.4766	0.5031	0.9245	0.027*	
C25	0.39610 (17)	0.46986 (15)	0.81225 (12)	0.0181 (4)	
H25A	0.4361	0.5070	0.7898	0.022*	
C26	0.38700 (16)	0.34037 (14)	0.64731 (12)	0.0147 (4)	
C27	0.46538 (17)	0.29877 (15)	0.70808 (13)	0.0195 (5)	
H27A	0.4705	0.3108	0.7604	0.023*	
C28	0.53603 (19)	0.23968 (16)	0.69202 (15)	0.0269 (5)	
H28A	0.5885	0.2106	0.7335	0.032*	
C29	0.53046 (19)	0.22288 (16)	0.61622 (15)	0.0285 (6)	
H29A	0.5793	0.1828	0.6056	0.034*	
C30	0.4533 (2)	0.26483 (16)	0.55562 (14)	0.0272 (5)	
H30A	0.4499	0.2541	0.5035	0.033*	
C31	0.38112 (18)	0.32224 (16)	0.57094 (12)	0.0216 (5)	
H31A	0.3273	0.3494	0.5292	0.026*	
C32	0.31737 (17)	0.52613 (14)	0.63571 (11)	0.0170 (4)	
C33	0.24358 (19)	0.59455 (16)	0.62971 (13)	0.0246 (5)	
H33A	0.1800	0.5811	0.6393	0.029*	
C34	0.2629 (2)	0.68252 (17)	0.60975 (15)	0.0343 (6)	
H34A	0.2128	0.7291	0.6063	0.041*	
C35	0.3542 (2)	0.70211 (18)	0.59500 (15)	0.0372 (7)	
H35A	0.3667	0.7619	0.5807	0.045*	
C36	0.4276 (2)	0.63505 (18)	0.60101 (15)	0.0340 (6)	
H36A	0.4910	0.6490	0.5913	0.041*	
C37	0.40962 (19)	0.54707 (16)	0.62126 (13)	0.0245 (5)	
H37A	0.4606	0.5012	0.6252	0.029*	
Cl1	0.26132 (6)	0.38380 (5)	0.35916 (4)	0.04137 (18)	
Cl2	0.32029 (5)	0.57549 (5)	0.36247 (4)	0.04004 (17)	
C38	0.2329 (3)	0.4967 (2)	0.3772 (3)	0.0854 (16)	
H38B	0.1610	0.5118	0.3428	0.102*	
H38A	0.2337	0.5015	0.4316	0.102*	

### Atomic displacement parameters (Å^2^)

**Table:**

	*U*^11^	*U*^22^	*U*^33^	*U*^12^	*U*^13^	*U*^23^
Cu1	0.01215 (14)	0.01202 (14)	0.01490 (13)	0.00153 (10)	0.00195 (10)	0.00055 (10)
P1	0.0126 (3)	0.0118 (3)	0.0126 (2)	0.0010 (2)	0.0035 (2)	0.00036 (19)
P2	0.0116 (3)	0.0140 (3)	0.0135 (2)	0.0009 (2)	0.0027 (2)	−0.0001 (2)
O1	0.0134 (7)	0.0150 (7)	0.0178 (7)	0.0033 (6)	0.0065 (6)	0.0027 (6)
O2	0.0168 (8)	0.0157 (7)	0.0163 (7)	0.0029 (6)	0.0070 (6)	0.0035 (6)
C1	0.0108 (10)	0.0102 (10)	0.0142 (9)	−0.0025 (8)	0.0021 (8)	−0.0034 (8)
C2	0.0128 (10)	0.0138 (10)	0.0161 (10)	0.0008 (8)	0.0071 (8)	0.0040 (8)
C3	0.0175 (11)	0.0155 (11)	0.0178 (10)	0.0000 (9)	0.0052 (8)	−0.0012 (8)
C4	0.0169 (11)	0.0255 (12)	0.0172 (10)	0.0022 (9)	0.0010 (9)	0.0031 (9)
C5	0.0147 (11)	0.0189 (11)	0.0289 (11)	0.0047 (9)	0.0079 (9)	0.0070 (9)
C6	0.0205 (11)	0.0160 (11)	0.0246 (11)	0.0016 (9)	0.0119 (9)	−0.0007 (9)
C7	0.0150 (10)	0.0173 (11)	0.0165 (10)	−0.0006 (9)	0.0070 (8)	0.0001 (8)
C8	0.0151 (10)	0.0170 (11)	0.0121 (9)	−0.0026 (9)	0.0038 (8)	−0.0031 (8)
C9	0.0204 (11)	0.0182 (11)	0.0201 (10)	−0.0006 (9)	0.0092 (9)	−0.0009 (9)
C10	0.0322 (14)	0.0218 (12)	0.0230 (11)	−0.0067 (10)	0.0131 (10)	0.0004 (9)
C11	0.0248 (13)	0.0285 (13)	0.0248 (12)	−0.0102 (10)	0.0148 (10)	−0.0090 (10)
C12	0.0174 (12)	0.0282 (13)	0.0299 (12)	−0.0016 (10)	0.0103 (10)	−0.0094 (10)
C13	0.0183 (11)	0.0191 (12)	0.0223 (11)	−0.0003 (9)	0.0077 (9)	−0.0035 (9)
C14	0.0131 (10)	0.0143 (10)	0.0144 (9)	0.0040 (8)	0.0039 (8)	−0.0001 (8)
C15	0.0184 (11)	0.0163 (11)	0.0170 (10)	0.0003 (9)	0.0032 (8)	0.0023 (8)
C16	0.0226 (12)	0.0179 (12)	0.0201 (11)	−0.0022 (9)	0.0001 (9)	−0.0008 (9)
C17	0.0293 (13)	0.0243 (12)	0.0140 (10)	0.0026 (10)	0.0012 (9)	0.0003 (9)
C18	0.0319 (13)	0.0220 (12)	0.0197 (11)	−0.0023 (10)	0.0095 (10)	0.0040 (9)
C19	0.0186 (11)	0.0171 (11)	0.0182 (10)	−0.0012 (9)	0.0063 (9)	−0.0007 (8)
C20	0.0142 (10)	0.0146 (11)	0.0157 (10)	0.0025 (8)	0.0052 (8)	−0.0010 (8)
C21	0.0191 (11)	0.0214 (12)	0.0216 (11)	−0.0034 (9)	0.0083 (9)	−0.0048 (9)
C22	0.0282 (13)	0.0256 (13)	0.0242 (11)	−0.0035 (10)	0.0162 (10)	−0.0003 (9)
C23	0.0270 (13)	0.0261 (13)	0.0158 (10)	0.0015 (10)	0.0087 (9)	−0.0012 (9)
C24	0.0204 (12)	0.0251 (12)	0.0196 (10)	−0.0029 (10)	0.0049 (9)	−0.0040 (9)
C25	0.0160 (11)	0.0211 (12)	0.0174 (10)	−0.0002 (9)	0.0056 (8)	0.0007 (9)
C26	0.0147 (10)	0.0120 (10)	0.0193 (10)	−0.0003 (8)	0.0079 (8)	−0.0001 (8)
C27	0.0161 (11)	0.0199 (12)	0.0210 (10)	0.0019 (9)	0.0040 (9)	0.0003 (9)
C28	0.0171 (12)	0.0220 (12)	0.0395 (14)	0.0045 (10)	0.0066 (10)	0.0019 (10)
C29	0.0240 (13)	0.0196 (12)	0.0497 (15)	0.0025 (10)	0.0226 (12)	−0.0036 (11)
C30	0.0363 (14)	0.0220 (13)	0.0315 (13)	−0.0033 (11)	0.0221 (11)	−0.0058 (10)
C31	0.0258 (12)	0.0206 (12)	0.0192 (10)	0.0007 (10)	0.0088 (9)	0.0020 (9)
C32	0.0178 (11)	0.0154 (11)	0.0144 (9)	−0.0003 (9)	0.0007 (8)	−0.0005 (8)
C33	0.0194 (12)	0.0204 (12)	0.0273 (12)	0.0023 (10)	−0.0013 (9)	−0.0004 (10)
C34	0.0358 (15)	0.0176 (13)	0.0365 (14)	0.0076 (11)	−0.0056 (11)	0.0019 (11)
C35	0.0493 (17)	0.0201 (13)	0.0329 (14)	−0.0060 (12)	0.0012 (12)	0.0103 (11)
C36	0.0391 (16)	0.0296 (14)	0.0358 (14)	−0.0068 (12)	0.0158 (12)	0.0087 (11)
C37	0.0259 (13)	0.0227 (12)	0.0254 (11)	0.0005 (10)	0.0091 (10)	0.0048 (10)
Cl1	0.0484 (4)	0.0482 (4)	0.0329 (3)	−0.0163 (3)	0.0207 (3)	0.0031 (3)
Cl2	0.0320 (4)	0.0366 (4)	0.0549 (4)	0.0016 (3)	0.0188 (3)	−0.0048 (3)
C38	0.069 (3)	0.053 (2)	0.173 (4)	0.033 (2)	0.092 (3)	0.070 (3)

### Geometric parameters (Å, º)

**Table:**

Cu1—O2	2.0794 (14)	C17—C18	1.382 (3)
Cu1—O1	2.1099 (14)	C17—H17A	0.9500
Cu1—P1	2.2213 (6)	C18—C19	1.391 (3)
Cu1—P2	2.2281 (6)	C18—H18A	0.9500
P1—C14	1.831 (2)	C19—H19A	0.9500
P1—C2	1.832 (2)	C20—C21	1.394 (3)
P1—C8	1.835 (2)	C20—C25	1.398 (3)
P2—C20	1.819 (2)	C21—C22	1.388 (3)
P2—C26	1.827 (2)	C21—H21A	0.9500
P2—C32	1.828 (2)	C22—C23	1.385 (3)
O1—C1	1.254 (2)	C22—H22A	0.9500
O2—C1^i^	1.254 (2)	C23—C24	1.382 (3)
C1—O2^i^	1.254 (2)	C23—H23A	0.9500
C1—C1^i^	1.568 (4)	C24—C25	1.387 (3)
C2—C3	1.396 (3)	C24—H24A	0.9500
C2—C7	1.396 (3)	C25—H25A	0.9500
C3—C4	1.384 (3)	C26—C31	1.394 (3)
C3—H3A	0.9500	C26—C27	1.395 (3)
C4—C5	1.387 (3)	C27—C28	1.390 (3)
C4—H4A	0.9500	C27—H27A	0.9500
C5—C6	1.390 (3)	C28—C29	1.381 (3)
C5—H5A	0.9500	C28—H28A	0.9500
C6—C7	1.383 (3)	C29—C30	1.388 (4)
C6—H6A	0.9500	C29—H29A	0.9500
C7—H7A	0.9500	C30—C31	1.384 (3)
C8—C13	1.394 (3)	C30—H30A	0.9500
C8—C9	1.396 (3)	C31—H31A	0.9500
C9—C10	1.389 (3)	C32—C37	1.388 (3)
C9—H9A	0.9500	C32—C33	1.394 (3)
C10—C11	1.383 (3)	C33—C34	1.393 (4)
C10—H10A	0.9500	C33—H33A	0.9500
C11—C12	1.388 (3)	C34—C35	1.374 (4)
C11—H11A	0.9500	C34—H34A	0.9500
C12—C13	1.386 (3)	C35—C36	1.376 (4)
C12—H12A	0.9500	C35—H35A	0.9500
C13—H13A	0.9500	C36—C37	1.390 (3)
C14—C15	1.392 (3)	C36—H36A	0.9500
C14—C19	1.397 (3)	C37—H37A	0.9500
C15—C16	1.388 (3)	Cl1—C38	1.761 (4)
C15—H15A	0.9500	Cl2—C38	1.737 (3)
C16—C17	1.384 (3)	C38—H38B	0.9900
C16—H16A	0.9500	C38—H38A	0.9900
			
O2—Cu1—O1	80.57 (5)	C18—C17—H17A	119.9
O2—Cu1—P1	111.21 (4)	C16—C17—H17A	119.9
O1—Cu1—P1	111.92 (4)	C17—C18—C19	120.2 (2)
O2—Cu1—P2	116.46 (4)	C17—C18—H18A	119.9
O1—Cu1—P2	100.25 (4)	C19—C18—H18A	119.9
P1—Cu1—P2	125.72 (2)	C18—C19—C14	119.9 (2)
C14—P1—C2	103.61 (9)	C18—C19—H19A	120.0
C14—P1—C8	102.50 (9)	C14—C19—H19A	120.0
C2—P1—C8	102.30 (9)	C21—C20—C25	119.10 (19)
C14—P1—Cu1	114.07 (7)	C21—C20—P2	118.78 (16)
C2—P1—Cu1	118.44 (7)	C25—C20—P2	122.08 (16)
C8—P1—Cu1	113.96 (7)	C22—C21—C20	120.3 (2)
C20—P2—C26	103.91 (9)	C22—C21—H21A	119.9
C20—P2—C32	103.18 (9)	C20—C21—H21A	119.9
C26—P2—C32	103.90 (10)	C23—C22—C21	120.2 (2)
C20—P2—Cu1	120.45 (7)	C23—C22—H22A	119.9
C26—P2—Cu1	110.67 (7)	C21—C22—H22A	119.9
C32—P2—Cu1	113.10 (7)	C24—C23—C22	119.9 (2)
C1—O1—Cu1	112.31 (12)	C24—C23—H23A	120.0
C1^i^—O2—Cu1	112.97 (12)	C22—C23—H23A	120.0
O2^i^—C1—O1	125.87 (18)	C23—C24—C25	120.3 (2)
O2^i^—C1—C1^i^	117.4 (2)	C23—C24—H24A	119.8
O1—C1—C1^i^	116.8 (2)	C25—C24—H24A	119.8
C3—C2—C7	118.68 (19)	C24—C25—C20	120.1 (2)
C3—C2—P1	118.08 (16)	C24—C25—H25A	119.9
C7—C2—P1	123.14 (16)	C20—C25—H25A	119.9
C4—C3—C2	120.7 (2)	C31—C26—C27	119.2 (2)
C4—C3—H3A	119.7	C31—C26—P2	116.21 (16)
C2—C3—H3A	119.7	C27—C26—P2	124.47 (16)
C3—C4—C5	120.2 (2)	C28—C27—C26	120.0 (2)
C3—C4—H4A	119.9	C28—C27—H27A	120.0
C5—C4—H4A	119.9	C26—C27—H27A	120.0
C4—C5—C6	119.5 (2)	C29—C28—C27	120.5 (2)
C4—C5—H5A	120.2	C29—C28—H28A	119.7
C6—C5—H5A	120.2	C27—C28—H28A	119.7
C7—C6—C5	120.3 (2)	C28—C29—C30	119.7 (2)
C7—C6—H6A	119.8	C28—C29—H29A	120.1
C5—C6—H6A	119.8	C30—C29—H29A	120.1
C6—C7—C2	120.52 (19)	C31—C30—C29	120.2 (2)
C6—C7—H7A	119.7	C31—C30—H30A	119.9
C2—C7—H7A	119.7	C29—C30—H30A	119.9
C13—C8—C9	119.1 (2)	C30—C31—C26	120.4 (2)
C13—C8—P1	117.61 (16)	C30—C31—H31A	119.8
C9—C8—P1	123.27 (16)	C26—C31—H31A	119.8
C10—C9—C8	120.3 (2)	C37—C32—C33	119.0 (2)
C10—C9—H9A	119.9	C37—C32—P2	124.09 (17)
C8—C9—H9A	119.9	C33—C32—P2	116.90 (17)
C11—C10—C9	120.4 (2)	C34—C33—C32	120.2 (2)
C11—C10—H10A	119.8	C34—C33—H33A	119.9
C9—C10—H10A	119.8	C32—C33—H33A	119.9
C10—C11—C12	119.5 (2)	C35—C34—C33	120.2 (2)
C10—C11—H11A	120.2	C35—C34—H34A	119.9
C12—C11—H11A	120.2	C33—C34—H34A	119.9
C13—C12—C11	120.5 (2)	C34—C35—C36	120.0 (2)
C13—C12—H12A	119.7	C34—C35—H35A	120.0
C11—C12—H12A	119.7	C36—C35—H35A	120.0
C12—C13—C8	120.2 (2)	C35—C36—C37	120.4 (3)
C12—C13—H13A	119.9	C35—C36—H36A	119.8
C8—C13—H13A	119.9	C37—C36—H36A	119.8
C15—C14—C19	119.30 (19)	C32—C37—C36	120.2 (2)
C15—C14—P1	122.34 (16)	C32—C37—H37A	119.9
C19—C14—P1	118.36 (16)	C36—C37—H37A	119.9
C16—C15—C14	120.4 (2)	Cl2—C38—Cl1	113.68 (18)
C16—C15—H15A	119.8	Cl2—C38—H38B	108.8
C14—C15—H15A	119.8	Cl1—C38—H38B	108.8
C17—C16—C15	120.0 (2)	Cl2—C38—H38A	108.8
C17—C16—H16A	120.0	Cl1—C38—H38A	108.8
C15—C16—H16A	120.0	H38B—C38—H38A	107.7
C18—C17—C16	120.1 (2)		
			
O2—Cu1—P1—C14	79.79 (8)	C8—P1—C14—C15	−26.7 (2)
O1—Cu1—P1—C14	−8.32 (9)	Cu1—P1—C14—C15	−150.37 (15)
P2—Cu1—P1—C14	−130.02 (7)	C2—P1—C14—C19	−100.01 (17)
O2—Cu1—P1—C2	−157.87 (8)	C8—P1—C14—C19	153.83 (17)
O1—Cu1—P1—C2	114.03 (8)	Cu1—P1—C14—C19	30.14 (18)
P2—Cu1—P1—C2	−7.68 (8)	C19—C14—C15—C16	−0.1 (3)
O2—Cu1—P1—C8	−37.48 (8)	P1—C14—C15—C16	−179.54 (17)
O1—Cu1—P1—C8	−125.58 (8)	C14—C15—C16—C17	1.2 (3)
P2—Cu1—P1—C8	112.72 (7)	C15—C16—C17—C18	−1.1 (4)
O2—Cu1—P2—C20	61.84 (9)	C16—C17—C18—C19	−0.2 (4)
O1—Cu1—P2—C20	146.33 (9)	C17—C18—C19—C14	1.4 (3)
P1—Cu1—P2—C20	−86.99 (8)	C15—C14—C19—C18	−1.2 (3)
O2—Cu1—P2—C26	−176.83 (8)	P1—C14—C19—C18	178.27 (17)
O1—Cu1—P2—C26	−92.34 (8)	C26—P2—C20—C21	−102.92 (18)
P1—Cu1—P2—C26	34.34 (8)	C32—P2—C20—C21	148.89 (17)
O2—Cu1—P2—C32	−60.73 (9)	Cu1—P2—C20—C21	21.7 (2)
O1—Cu1—P2—C32	23.77 (9)	C26—P2—C20—C25	74.82 (19)
P1—Cu1—P2—C32	150.44 (8)	C32—P2—C20—C25	−33.4 (2)
O2—Cu1—O1—C1	−1.49 (13)	Cu1—P2—C20—C25	−160.60 (15)
P1—Cu1—O1—C1	107.70 (13)	C25—C20—C21—C22	−1.3 (3)
P2—Cu1—O1—C1	−116.89 (13)	P2—C20—C21—C22	176.49 (18)
O1—Cu1—O2—C1^i^	1.51 (13)	C20—C21—C22—C23	0.3 (3)
P1—Cu1—O2—C1^i^	−108.46 (13)	C21—C22—C23—C24	0.7 (4)
P2—Cu1—O2—C1^i^	98.33 (13)	C22—C23—C24—C25	−0.6 (4)
Cu1—O1—C1—O2^i^	−178.72 (16)	C23—C24—C25—C20	−0.5 (3)
Cu1—O1—C1—C1^i^	1.2 (3)	C21—C20—C25—C24	1.4 (3)
C14—P1—C2—C3	171.60 (16)	P2—C20—C25—C24	−176.32 (17)
C8—P1—C2—C3	−82.10 (18)	C20—P2—C26—C31	177.94 (17)
Cu1—P1—C2—C3	44.12 (18)	C32—P2—C26—C31	−74.41 (18)
C14—P1—C2—C7	−12.1 (2)	Cu1—P2—C26—C31	47.28 (18)
C8—P1—C2—C7	94.24 (18)	C20—P2—C26—C27	2.3 (2)
Cu1—P1—C2—C7	−139.54 (15)	C32—P2—C26—C27	109.95 (19)
C7—C2—C3—C4	−2.0 (3)	Cu1—P2—C26—C27	−128.36 (18)
P1—C2—C3—C4	174.51 (17)	C31—C26—C27—C28	−0.4 (3)
C2—C3—C4—C5	0.9 (3)	P2—C26—C27—C28	175.17 (17)
C3—C4—C5—C6	1.0 (3)	C26—C27—C28—C29	1.1 (4)
C4—C5—C6—C7	−1.6 (3)	C27—C28—C29—C30	−0.5 (4)
C5—C6—C7—C2	0.5 (3)	C28—C29—C30—C31	−0.9 (4)
C3—C2—C7—C6	1.3 (3)	C29—C30—C31—C26	1.7 (4)
P1—C2—C7—C6	−174.98 (16)	C27—C26—C31—C30	−1.1 (3)
C14—P1—C8—C13	−75.12 (17)	P2—C26—C31—C30	−176.95 (18)
C2—P1—C8—C13	177.72 (16)	C20—P2—C32—C37	101.43 (19)
Cu1—P1—C8—C13	48.64 (17)	C26—P2—C32—C37	−6.8 (2)
C14—P1—C8—C9	104.56 (18)	Cu1—P2—C32—C37	−126.83 (17)
C2—P1—C8—C9	−2.60 (19)	C20—P2—C32—C33	−80.39 (18)
Cu1—P1—C8—C9	−131.68 (16)	C26—P2—C32—C33	171.41 (16)
C13—C8—C9—C10	0.2 (3)	Cu1—P2—C32—C33	51.35 (18)
P1—C8—C9—C10	−179.45 (17)	C37—C32—C33—C34	−0.2 (3)
C8—C9—C10—C11	0.9 (3)	P2—C32—C33—C34	−178.49 (17)
C9—C10—C11—C12	−0.8 (3)	C32—C33—C34—C35	0.7 (4)
C10—C11—C12—C13	−0.5 (3)	C33—C34—C35—C36	−0.9 (4)
C11—C12—C13—C8	1.7 (3)	C34—C35—C36—C37	0.6 (4)
C9—C8—C13—C12	−1.5 (3)	C33—C32—C37—C36	−0.1 (3)
P1—C8—C13—C12	178.17 (16)	P2—C32—C37—C36	178.04 (18)
C2—P1—C14—C15	79.48 (19)	C35—C36—C37—C32	−0.1 (4)

Symmetry code: (i) −*x*, −*y*+1, −*z*+1.
