# μ-Oxalato-bis­[bis­(triphenyl­phosphine)copper(I)] dichloro­methane disolvate. Corrigendum

**DOI:** 10.1107/S1600536814019692

**Published:** 2014-09-13

**Authors:** Andrew D. Royappa, James A. Golen, Arnold L. Rheingold, A. Timothy Royappa

**Affiliations:** a Department of Chemistry, University of West Florida, 11000 University Parkway, Pensacola, FL 32514, USA; b Department of Chemistry, University of Massachusetts Dartmouth, 285 Old Westport Road, North Dartmouth, MA 02747, USA; c Department of Chemistry, University of California, San Diego, Urey Hall 5128, mail code 0358, 9500 Gilman Drive, La Jolla, CA 92093, USA

## Abstract

An erroneous claim in the paper by Royappa *et al.* [*Acta Cryst.* (2013), E**69**, m126] is corrected and a reference added for a previously published report of a closely related structure.

## Abstract 2

Corrigendum to *Acta Cryst.* (2013), E**69**, m126.

In the paper by Royappa *et al.* (2013 ▶), the authors claimed ‘To date, no examples of copper(I) oxalate compounds containing triphenylphosphine ligands coordinated through the phosphorus atoms to the metal centers have been structurally characterized’.

However, the authors were unaware of a previous report (Jakob *et al.*, 2010 ▶) on the structure of (PPh_3_)_2_Cu(C_2_O_4_)Cu(PPh_3_)_2_ with a different number of dichloromethane solvent molecules. The authors sincerely regret this unintentional oversight.
